# Supplementary material for: Gentle and fast all-atom model refinement to cryo-EM densities via a maximum likelihood approach
Source: PLoS Comput Biol. 2023 Jul 31;19(7):e1011255. doi: 10.1371/journal.pcbi.1011255 (PMC10427019; doi:10.1371/journal.pcbi.1011255)
Supplement: S1 Appendix — Derivation of relative entropy measures from Poisson noise assumption, grid scattering assumptions, model density gradients and similarity measure definitions. (PDF) [file pcbi.1011255.s001.pdf]

Supporting Information S1 Appendix

Gentle and fast all-atom model refinement to cryo-EM densities via a maximum likelihood approach

Christian Blau, Linnea Yvonnesdotter, Erik Lindahl*

* erik.lindahl@dbb.su.se

1 Potential derivation details

1.1 Derivation of relative entropy potential from Poisson noise assumption

By using Boltzmann inversion for the total likelihood

$$\log p\left(\rho|\rho^s(\vec{x})\right) = -\frac{1}{k_B T} U_{\mathrm{fit}}(\vec{x},\rho) + c\,. \tag{1}$$

and the assumption that electrons scatter independently in voxels

$$p(\rho|\rho^s(\vec{x})) = \prod_{v\in\mathrm{voxels}} p(\rho_v|\rho_v^s) \tag{2}$$

$$\log p(\rho|\rho^s(\vec{x})) = \sum_{v\in\mathrm{voxels}} \log p(\rho_v|\rho_v^s)\,, \tag{3}$$

as well as a Poisson distribution in each voxel with distribution parameters $\mathrm{Pois}(N\rho_v^s, r\rho_v)$, where $r\rho_v =: \hat{\rho_v}$ represents an interaction count,

$$p(\rho_v|\rho_v^s) = (N\rho_v^s)^{\hat{\rho_v}} \exp(-N\rho_v^s)/\hat{\rho_v}! \tag{4}$$

$$\log p(\rho_v|\rho_v^s) = \hat{\rho_v} \log\left(N\rho_v^s\right) - N\rho_v^s - \log(\hat{\rho_v}!)\,. \tag{5}$$

we obtain the log-likelihood

$$\log p(\rho|\rho^s(\vec{x})) = \sum_{v\in\mathrm{voxels}} -N\rho_v^s + \hat{\rho_v}\log N + \hat{\rho_v}\log\rho_v^s - \log\hat{\rho_v}!\,. \tag{6}$$

With the Stirling approximation $\log\hat{\rho_v}! \approx \hat{\rho_v}\log\hat{\rho_v} - \hat{\rho_v}$, assuming $\hat{\rho_v}$ is sufficiently large, this is simplified to

$$\log p(\rho|\rho^s) = -\sum_{v\in\mathrm{voxels}} N\rho_v^s + \sum_{v\in\mathrm{voxels}} \hat{\rho_v}(\log N + 1) + \sum_{v\in\mathrm{voxels}} \hat{\rho_v}\log\rho_v^s - \hat{\rho_v}\log\hat{\rho_v} \tag{7}$$

According to eq. (1), similarity scores may be rescaled up to a constant which means that the term $\sum_v \hat{\rho_v}(\log N + 1)$ that does not depend on the model coordinates may be dropped from the right hand side of eq. (7). When the summed electron scattering probability is constant, e.g., when generating the model density from a sum of Gaussians as used below, the sum $\sum_v N\rho_v^s$ is also constant. With this regauging we obtain

$$\log p(\rho|\rho^s(\vec{x})) = \sum_{v \in \text{voxels}} \hat{\rho_v} \log \frac{\rho_v^s}{\hat{\rho_v}} + \text{const}\,. \tag{8}$$

1.1.1 Conditional posterior

The scaling parameter r for the simulated density above may be estimated from a set of coordinates as

$$p(r|\vec{x}, \rho) \propto \frac{1}{r} \exp\left(\sum_v r\rho_v \log \frac{\rho_v^s(\vec{x})}{r\rho_v}\right) \tag{9}$$

1.2 Derivation of the swapped relative-entropy potential from Dirichlet distribution assumption

To derive the fitting potential from eq. (5) in the main text, we apply the log,

$$\log p_I\left(\rho|\vec{x}, s\right) = \log\Gamma\left(\sum_v s\rho_v^s\right) - \sum_v \log\Gamma\left(s\rho_s^v\right) + \sum_v (s\rho_v^s - 1)\log\rho_v, \tag{10}$$

and use the fact that we normalize the simulated density to unity, $\sum_v \rho_v^s = 1$,

$$\log p_I\left(\rho|\vec{x}, s\right) = s\sum_v \rho_v^s \log\rho_v - \sum_v \log\rho_v - \sum_v \log\Gamma\left(s\rho_s^v\right), \tag{11}$$

Using Stirling's series,

$$\log p_I\left(\rho|\vec{x}, s\right) \approx s\sum_v \rho_v^s \log\rho_v - \sum_v \log\rho_v - \sum_v \left(\frac{1}{2}\log(2\pi) + \left(s\rho_s^v - \frac{1}{2}\right)\log s\rho_s^v - s\rho_s^v\right), \tag{12}$$

Skipping all constant terms, because the potential can be shifted arbitrarily, we obtain

$$\log p_I\left(\rho|\vec{x}, s\right) \approx s\sum_v \rho_v^s \log\rho_v - \sum_v \left(\left(s\rho_s^v - \frac{1}{2}\right)\log s\rho_s^v\right), \tag{13}$$

We assume that the second summand varies less than the first, which is guaranteed when the derivatives of $\log p_I$ at all voxels v with respect to ρ_v^s are smaller in the first summand than the second,

$$\log\rho_v > \log(s\rho_v^s) + 1 - \frac{1}{2s\rho_v^s} \tag{14}$$

$$\rho_v > s\rho_v^s \exp\left(1 - \frac{1}{2s\rho_v^s}\right). \tag{15}$$

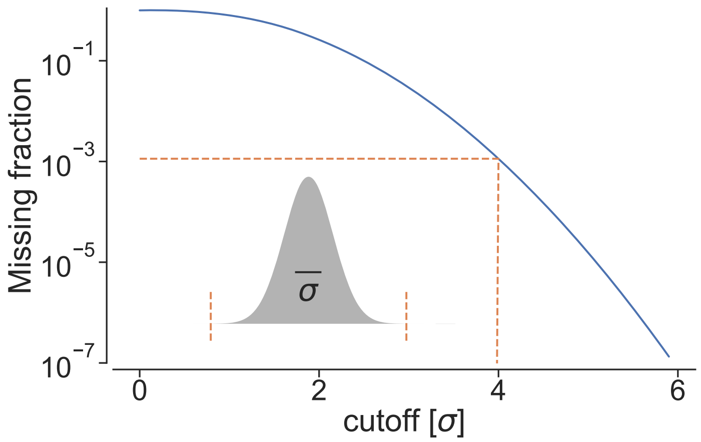

Fig A: Missing contribution from three-dimensional Gaussian mass density due to cutoffs at different σ. Orange lines indicate a cutoff at 4σ, as used in all refinements in this publication.

Leading to the approximation

$$\log p_I\left(\rho|\vec{x}, s\right) \approx s \sum_v \rho_v^s \log \rho_v . \tag{16}$$

1.2.1 Conditional posterior

Using the approximations above, and a prior $p(s) \propto s^{-1}$, s might be estimated from a distribution of $\vec{x}$

$$p(s|\vec{x}, \rho) \propto \frac{1}{s} \exp\left(s \sum_v \rho_v^s(\vec{x}) \log \rho_v\right) \tag{17}$$

1.3 Re-gauging of the relative-entropy based potential

The scaling of the voxel values by a factor f multiplies the relative-entropy score with f and adds a constant offset in the similarity score which can be ignored if used as a potential:

$$\sum_{v \in \text{voxels}} f\rho_v \log \frac{\rho_v^s}{f\rho_v} = f \sum_{v \in \text{voxels}} \rho_v \log \frac{\rho_v^s}{\rho_v} - \underbrace{\rho_v \log f}_{\text{constant}} . \tag{18}$$

2 Gaussian spreading on a grid

To determine the contribution of an atom at coordinate r with an intensity A to the model density, we project a Gaussian at r onto the discrete grid used to describe cryo-EM densities by integration of the Gaussian over the voxel volume,

$$\rho_{\mathbf{v}} = \int_{x \in \mathbf{v}} A \frac{1}{\sqrt{2\pi}^3 \sigma^3} \exp\left[-\frac{(\mathbf{r} - \mathbf{x})^2}{2\sigma^2}\right] \tag{19}$$

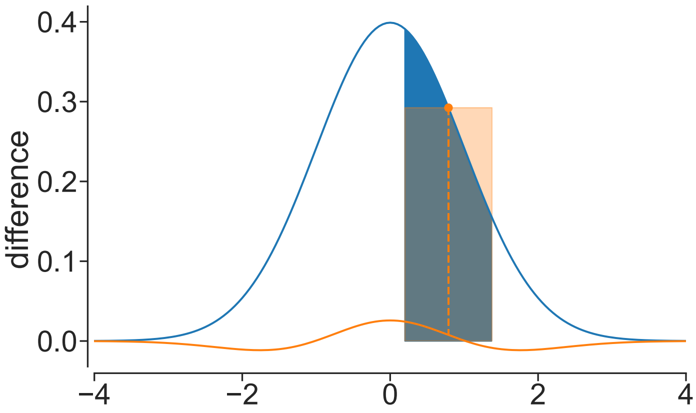

Fig B: Difference between integral over a voxel (blue area) of Gaussian function (blue line) and approximation by mid-point evaluation (orange) using a voxel size of $\sqrt{\log 4}\sigma$.

Two approximations speed up computation of these contributions in practice. First, we ignore contributions to voxels with their centers further away than 4σ from the atom, since this overall amounts to very little loss of atom density as shown in Fig A. Second, we approximate the integral in eq. (19) by a discrete Gauss transform. Fig B shows the difference of the Gauss transform to integrating over voxel density when applying the $\sigma = \delta/\sqrt{\log 4}$ discretisation criterion as suggested in the main text.

3 The model density gradient

Using the discrete Gauss transform on the voxel grid, the density spreading contributes to the force with the gradient of the model density,

$$\nabla_r \rho_v^{\mathrm{s}}(\mathbf{r}) = \sum_i -A_i \frac{(\mathbf{r_i} - \mathbf{v})}{\sigma} \frac{1}{\sqrt{2\pi}^3 \sigma^3} \exp[-\frac{(\mathbf{r_i} - \mathbf{v})^2}{2\sigma^2}]\,. \tag{20}$$

4 Explicit similarity measure definitions

The similarity measures over voxels v are defined for inner-product, cross-correlation, swapped-relative-entropy and normal relative-entropy respectively,

$$S_{\mathrm{ip}}\left(\rho, \rho^{\mathrm{s}}\right) := \frac{1}{N_{\mathrm{voxel}}} \sum_v \rho_v \rho_v^{\mathrm{s}} \tag{21}$$

$$S_{\mathrm{cc}}\left(\rho, \rho^{\mathrm{s}}\right) := \frac{\sum_v \left[(\rho_v - \bar{\rho})(\rho_v^{\mathrm{s}} - \bar{\rho}^{\mathrm{s}})\right]}{\sqrt{\sum_v (\rho_v - \bar{\rho})^2 \sum_v (\rho_v^{\mathrm{s}} - \bar{\rho}^{\mathrm{s}})^2}} \tag{22}$$

$$S_{\mathrm{re-swapped}}\left(\rho, \rho^{\mathrm{s}}\right) := \sum_{v, \rho_v > 0, \rho_v^{\mathrm{s}} > 0} \rho_v^{\mathrm{s}} \log(\rho_v) \tag{23}$$

$$S_{\mathrm{re}}\left(\rho, \rho^{\mathrm{s}}\right) := \sum_{v, \rho_v > 0, \rho_v^{\mathrm{s}} > 0} \rho_v [\log(\rho_v^{\mathrm{s}}) - \log(\rho_v)] \tag{24}$$

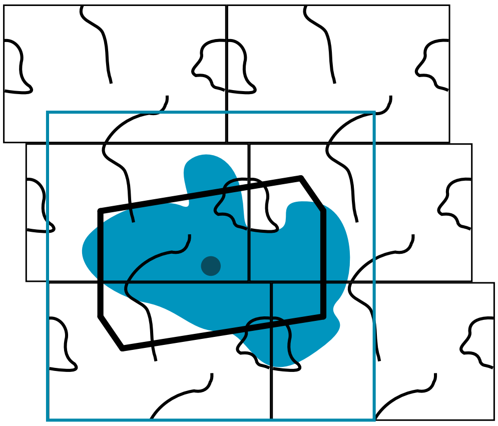

Fig C: When refining against a density (blue), only the periodic image of the atoms of the simulated molecules (black lines) is chosen that is closest to the center (blue dot) of the density boundary box (blue lines), effectively generating a Wigner-Seitz cell (thick black lines) of effective density influence around the density boundary box center.

5 Treatment of periodic boundary conditions

In the molecular dynamics simulation periodic boundary conditions are used. When multiple periodic images of the same atom are within the extent of the cryo-EM density voxel grid (Fig C), only the one closest to the density map center is contributing to the model density. This allows refinement simulations without any restrictions on the respective size of simulation boxes and voxel grids and easy treatment with pressure coupling algorithms that change the size of the simulation box. In a few special setups, a situation may occur where different periodic images of the same molecule contribute to density based potential, thus "pulling" the molecule apart, which is easily avoided by either increasing the simulation box size or reducing the voxel grid, respectively.
