# Supplementary material for: Gentle and fast all-atom model refinement to cryo-EM densities via a maximum likelihood approach
Source: PLoS Comput Biol. 2023 Jul 31;19(7):e1011255. doi: 10.1371/journal.pcbi.1011255 (PMC10427019; doi:10.1371/journal.pcbi.1011255)
Supplement: S1 Table — Scattering amplitudes for common atoms in biological molecules at 150keV, derived from Appendix C in Ref. [31]. (PDF) [file pcbi.1011255.s002.pdf]

Element	cross section
H	0.04448
C	1
N	0.78061
O	0.62773
P	4.79207
S	4.24146
