# Supplementary material for: Gentle and fast all-atom model refinement to cryo-EM densities via a maximum likelihood approach
Source: PLoS Comput Biol. 2023 Jul 31;19(7):e1011255. doi: 10.1371/journal.pcbi.1011255 (PMC10427019; doi:10.1371/journal.pcbi.1011255)
Supplement: S2 Table — Heavy-atom RMSD [Å] at the final frame compared to conformation from which density was generated. (PDF) [file pcbi.1011255.s003.pdf]

replicate	1	2	3	4	5	6	7
inner-product	1.01	5.42	0.753	7.33	7.55	7.89	0.53
cross-correlation	3.96	6.41	5.86	5.92	7.32	3.85	3.93
relative-entropy-swapped	5.5	15.7	7.31	17.0	13.1	11.3	0.747
relative-entropy	2.07	4.21	0.732	7.19	2.06	2.38	3.55
