# Supplementary material for: Gentle and fast all-atom model refinement to cryo-EM densities via a maximum likelihood approach
Source: PLoS Comput Biol. 2023 Jul 31;19(7):e1011255. doi: 10.1371/journal.pcbi.1011255 (PMC10427019; doi:10.1371/journal.pcbi.1011255)
Supplement: S3 Table — Heavy-atom RMSD [Å] at the final frame compared to conformation from which density was generated. (PDF) [file pcbi.1011255.s004.pdf]

replicate	1	2	3	4	5	6	7
inner-product	4.05	23.3	12.2	12.5	22.4	7.83	0.832
cross-correlation	16.7	8.78	8.81	0.777	25.6	21.4	27.7
relative-entropy-swapped	50.2	33.2	26.8	56.7	62.2	43.5	74.0
relative-entropy	0.837	0.748	0.803	0.661	0.685	0.726	8.21
