# Supplementary material for: Gentle and fast all-atom model refinement to cryo-EM densities via a maximum likelihood approach
Source: PLoS Comput Biol. 2023 Jul 31;19(7):e1011255. doi: 10.1371/journal.pcbi.1011255 (PMC10427019; doi:10.1371/journal.pcbi.1011255)
Supplement: S4 Table — Heavy-atom RMSD [Å] from final simulation frames, as compared to PDB id 6V20. (PDF) [file pcbi.1011255.s005.pdf]

replicate	1	2	3
inner-product	0.967	0.871	0.926
cross-correlation	0.926	0.916	0.960
relative-entropy-swapped	0.908	0.924	0.937
relative-entropy	0.974	0.987	1.050
