# Supplementary material for: Gentle and fast all-atom model refinement to cryo-EM densities via a maximum likelihood approach
Source: PLoS Comput Biol. 2023 Jul 31;19(7):e1011255. doi: 10.1371/journal.pcbi.1011255 (PMC10427019; doi:10.1371/journal.pcbi.1011255)
Supplement: S5 Table — Properties calculated with phenix-1.18.2–3874, using molprobity, CABLAM, and EMRinger methods. All data reflect the final frame without any further geometry optimization. (PDF) [file pcbi.1011255.s006.pdf]

method	inner-prod.			cross-corr.			re-swapped			rel-entropy		
replicate	1	2	3	1	2	3	1	2	3	1	2	3
Ramachandran outliers (%)	1.03	0.81	1.25	1.03	1.10	1.03	0.66	1.25	1.17	1.69	1.69	1.61
Ramachandran favored	96.85	96.26	96.48	96.19	95.31	96.19	95.82	95.23	95.82	95.01	94.65	93.70
Rotamer outliers	3.49	3.29	2.81	3.29	3.20	3.68	4.75	3.29	4.55	5.14	5.43	7.27
C-beta deviations	91	93	99	110	107	125	107	97	93	115	99	133
Clashscore	0.58	1.21	0.82	0.92	1.26	1.41	0.92	1.41	1.31	1.31	0.92	1.21
RMS(bonds)	0.0376	0.0384	0.0377	0.0391	0.0384	0.0433	0.0362	0.0357	0.0352	0.0363	0.0368	0.0375
RMS(angles)	3.51	3.50	3.52	3.61	3.61	3.76	3.50	3.51	3.48	3.55	3.50	3.58
MolProbity score	1.30	1.48	1.33	1.43	1.56	1.56	1.58	1.60	1.65	1.74	1.70	1.91
CABLAM disfavored (¡5%)	5.3	5.6	5.5	5.4	5.8	5.5	5.5	7.0	5.2	7.0	6.2	7.2
CABLAM outlier (¡1.5%)	1.8	1.4	2.0	1.5	1.7	2.1	1.5	2.6	2.1	2.6	2.2	2.0
CABLAM CA geometry outlier (¡0.5)	0.44	0.37	0.52	0.59	0.52	0.37	0.66	0.59	0.59	0.44	0.81	0.74
EMRinger Score	6.03	6.06	6.49	6.13	6.04	6.41	5.96	5.75	5.42	5.03	5.64	5.40
