# Supplementary material for: Gentle and fast all-atom model refinement to cryo-EM densities via a maximum likelihood approach
Source: PLoS Comput Biol. 2023 Jul 31;19(7):e1011255. doi: 10.1371/journal.pcbi.1011255 (PMC10427019; doi:10.1371/journal.pcbi.1011255)
Supplement: S6 Table — Heavy-atom RMSD [Å] from final simulation frames, as compared to conformational state 3 of PDB id 5W0S. (PDF) [file pcbi.1011255.s007.pdf]

replicate	1	2	3
inner-product	1.36	1.39	1.41
cross-correlation	1.38	1.4	1.41
relative-entropy-swapped	1.48	1.47	1.49
relative-entropy	1.96	1.81	1.75
