# Supplementary material for: Gentle and fast all-atom model refinement to cryo-EM densities via a maximum likelihood approach
Source: PLoS Comput Biol. 2023 Jul 31;19(7):e1011255. doi: 10.1371/journal.pcbi.1011255 (PMC10427019; doi:10.1371/journal.pcbi.1011255)
Supplement: S7 Table — Properties calculated with phenix-1.18.2–3874, using molprobity, CABLAM, and EMRinger methods. All data reflect the final frame without any further geometry optimization. (PDF) [file pcbi.1011255.s008.pdf]

method	inner-prod.			cross-corr.			re-swapped			rel-entropy		
replicate	1	2	3	1	2	3	1	2	3	1	2	3
Ramachandran outliers	0.96	0.57	0.38	1.53	0.00	0.57	3.07	1.34	2.30	2.30	0.77	1.15
favored	94.83	94.83	95.40	93.49	95.59	94.83	89.08	91.38	92.53	89.08	92.34	93.68
Rotamer outliers	5.91	2.69	4.57	7.53	5.91	6.72	15.05	11.83	7.53	6.99	4.03	5.38
C-beta deviations	51	40	52	49	51	46	67	54	47	56	54	51
Clashscore	1.83	1.31	1.57	1.83	1.04	1.18	5.35	1.96	3.13	0.26	0.78	0.91
RMS(bonds)	0.0400	0.0377	0.0408	0.0398	0.0378	0.0365	0.0388	0.0375	0.0364	0.0373	0.0361	0.0347
RMS(angles)	3.56	3.56	3.63	3.62	3.53	3.57	3.83	3.70	3.61	3.70	3.64	3.63
MolProbity score	1.89	1.54	1.72	2.04	1.70	1.82	2.76	2.28	2.24	1.81	1.68	1.75
CABLAM disfavored (¡5%)	7.3	6.5	7.1	7.5	8.3	7.3	14.4	12.7	11.5	14.8	9.4	7.7
CABLAM outlier (¡1.5%)	1.7	1.3	1.9	2.3	2.7	2.1	7.1	4.4	4.4	5.6	3.1	2.3
CABLAM CA geometry outlier (¡0.5)	0.96	0.77	0.77	0.58	0.77	1.15	2.12	1.73	1.73	1.92	0.77	1.54
EMRinger Score	2.08	2.14	2.05	1.73	1.51	1.13	2.19	2.05	1.88	1.35	1.69	1.95
