# Supplementary figures and images for: Gentle and fast all-atom model refinement to cryo-EM densities via a maximum likelihood approach
Source: PLoS Comput Biol. 2023 Jul 31;19(7):e1011255. doi: 10.1371/journal.pcbi.1011255 (PMC10427019; doi:10.1371/journal.pcbi.1011255)

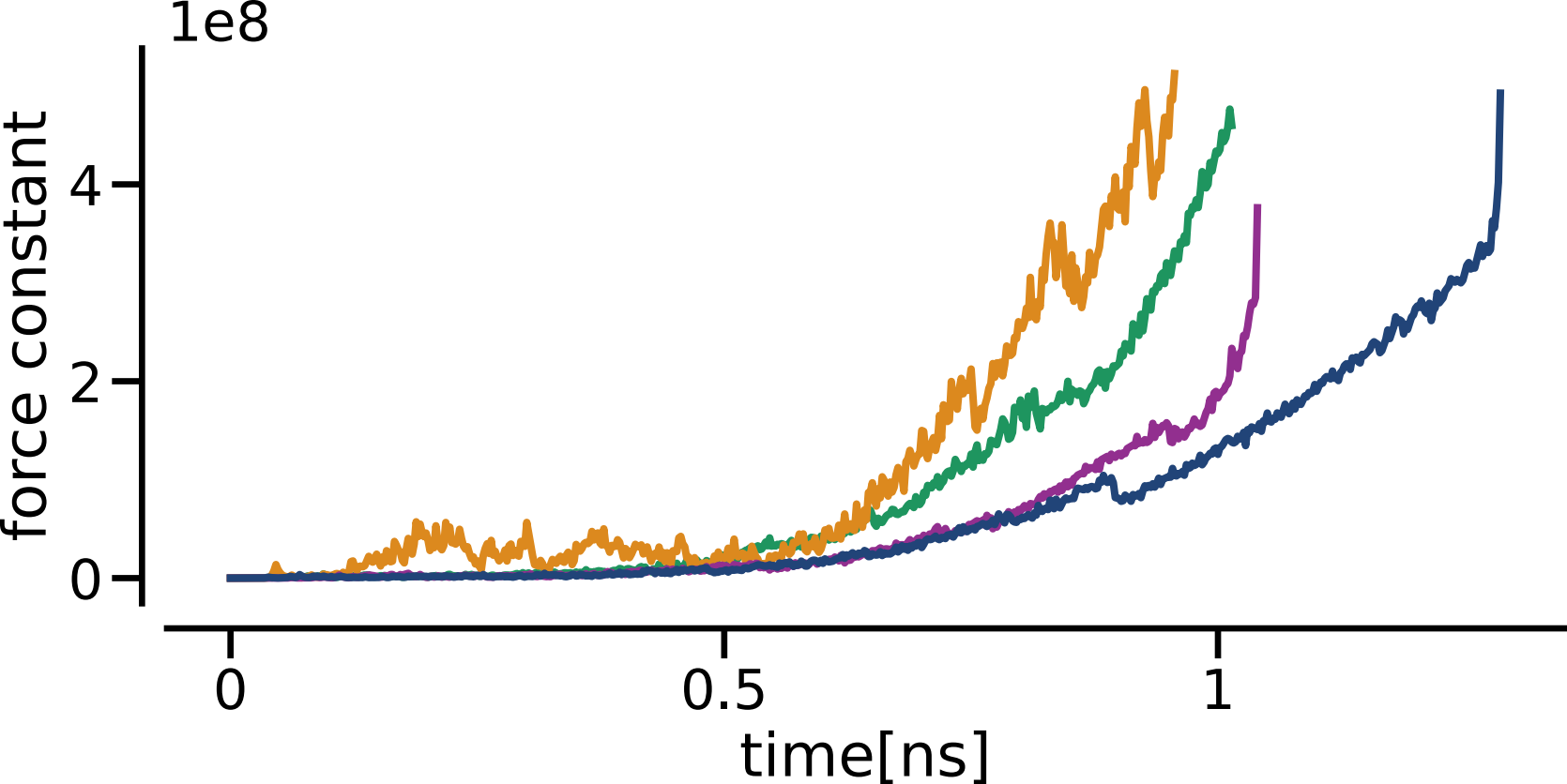

Supplement: S1 Fig — Evolution of force constant (arbitrary units) during aligned helix refinement for inner-product(purple), cross-correlation(ochre), relative-entropy(green) and relative-entropy-swapped (blue). (PDF) [file pcbi.1011255.s009.pdf]

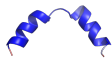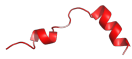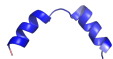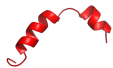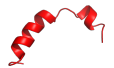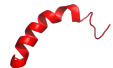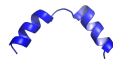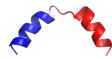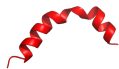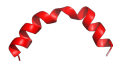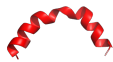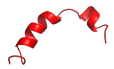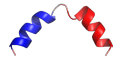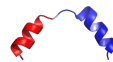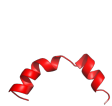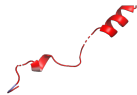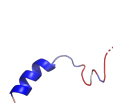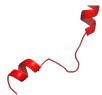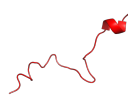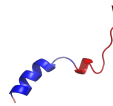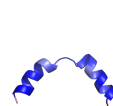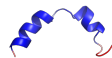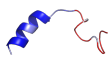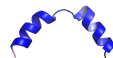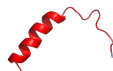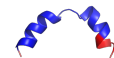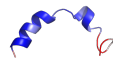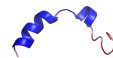

Supplement: S2 Fig — Simulations using adaptive force scaling from aligned starting conformation using inner-product, cross-correlation, relative-entropy swapped, and relative entropy (ordered top to bottom). (PDF) [file pcbi.1011255.s010.pdf]

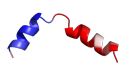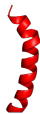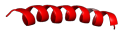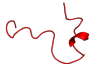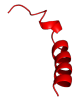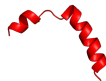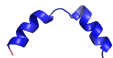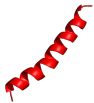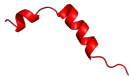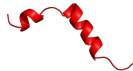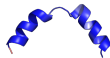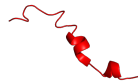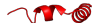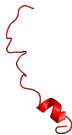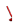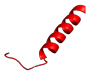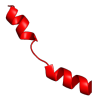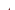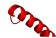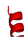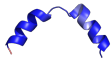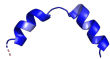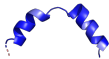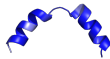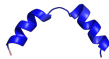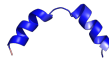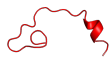

Supplement: S3 Fig — Simulations using adaptive force scaling from aligned starting conformation using inner-product, cross-correlation, relative-entropy swapped, and relative entropy (ordered top to bottom). (PDF) [file pcbi.1011255.s011.pdf]

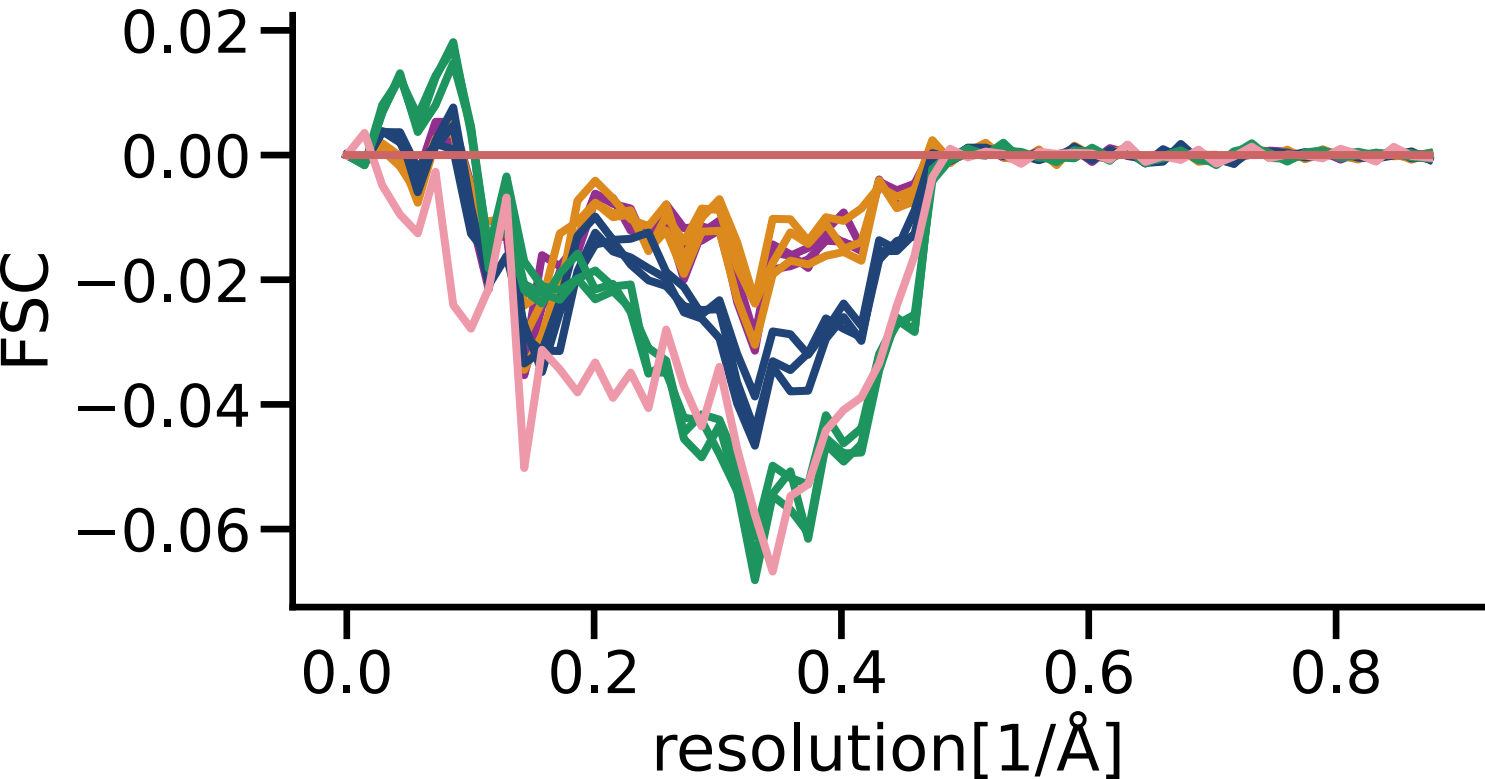

Supplement: S4 Fig — Difference in FSC to deposited Aldolase model for inner-product (purple), cross-correlation (ochre), swapped relative-entropy (blue) and relative-entropy (green) based on refinement final frames (solid). (PDF) [file pcbi.1011255.s012.pdf]

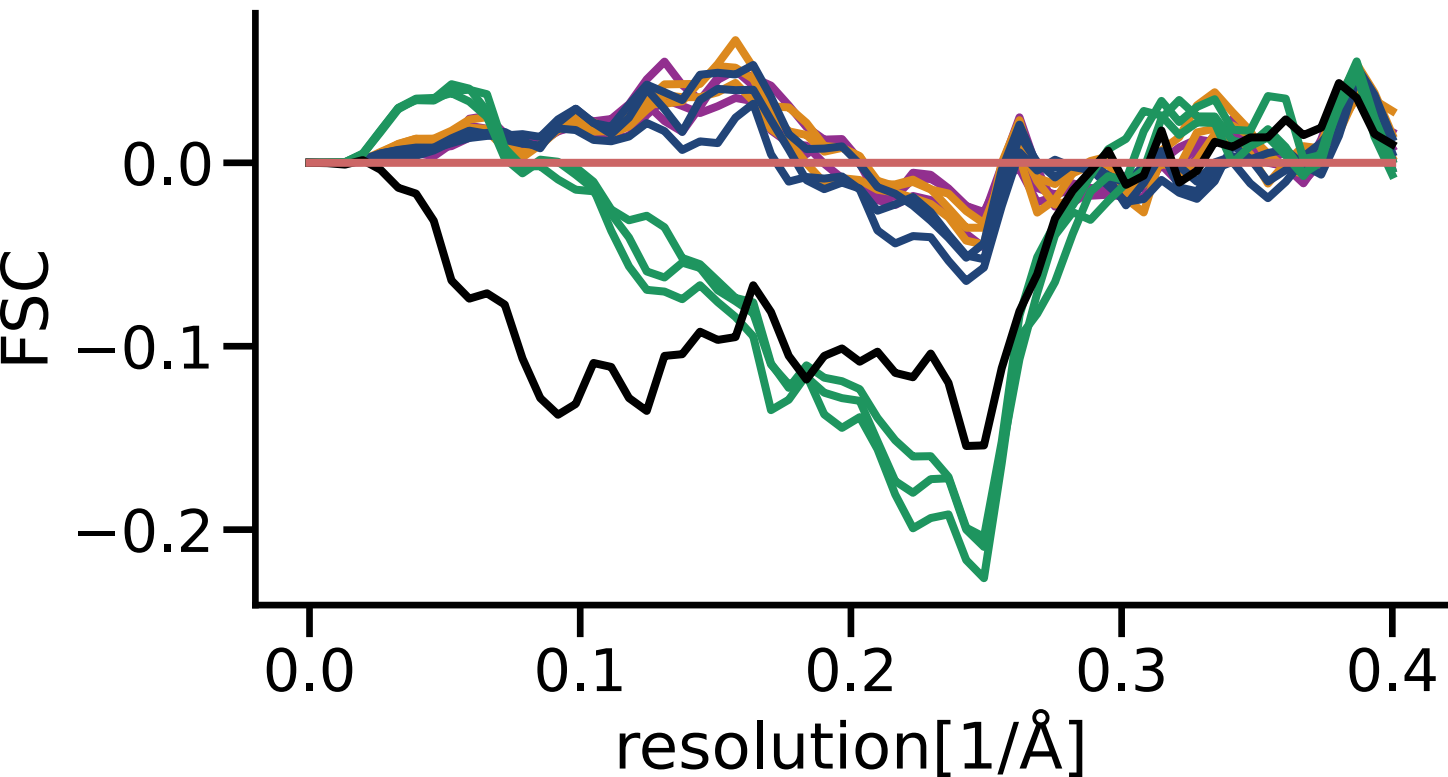

Supplement: S5 Fig — Difference in FSC to deposited GroEL model for inner-product (purple), cross-correlation (ochre), swapped relative-entropy (blue) and relative-entropy (green) based on refinement final frames (solid). (PDF) [file pcbi.1011255.s013.pdf]

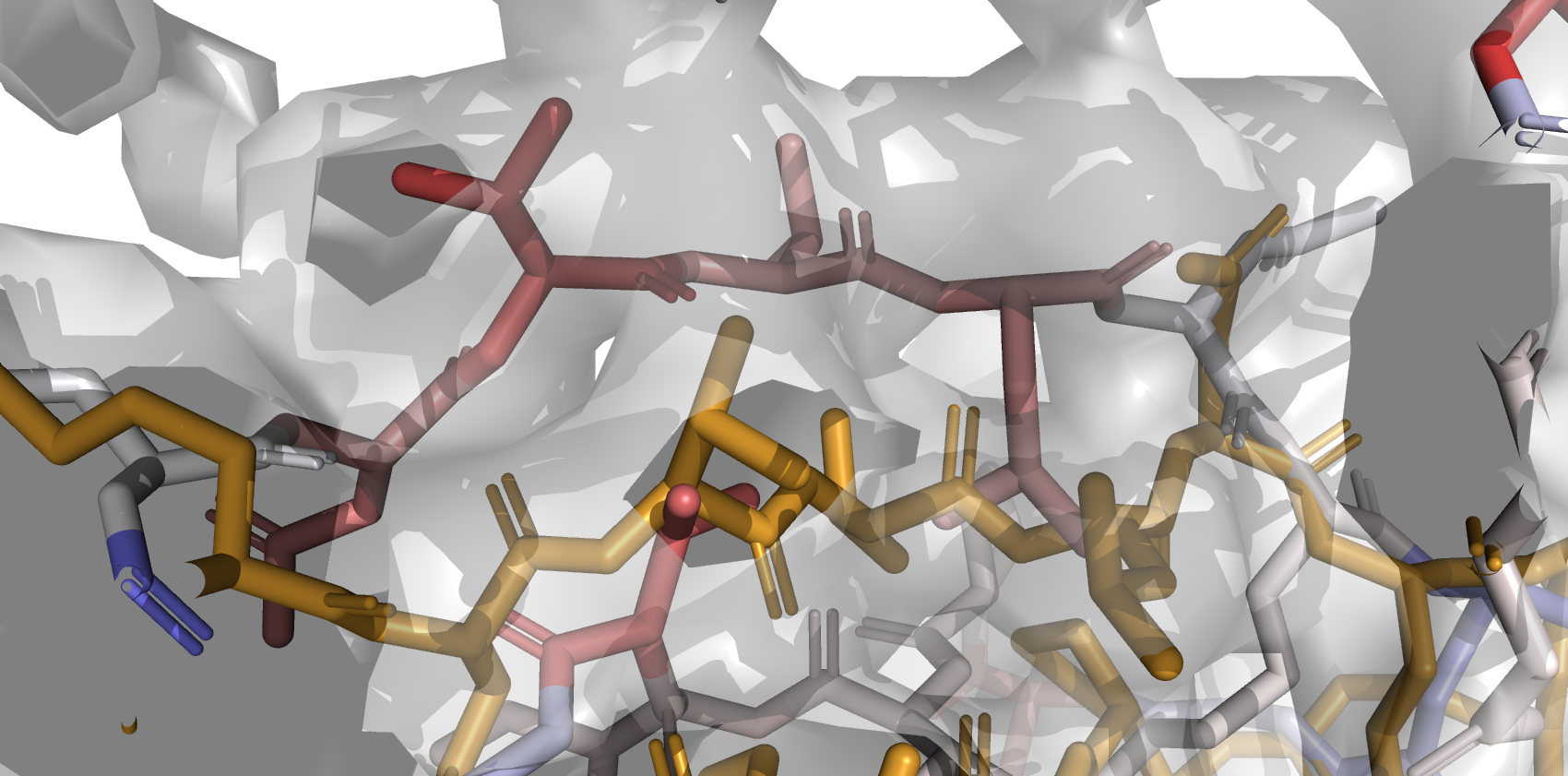

Supplement: S6 Fig — Relative entropy-based refinement (blue-red, according to RMSD to published model) can struggles in regions with extra density present in the map, whereas the cross-correlation based potential (ochre) adheres to the local minimum defined by the density. (TIF) [file pcbi.1011255.s014.tif]

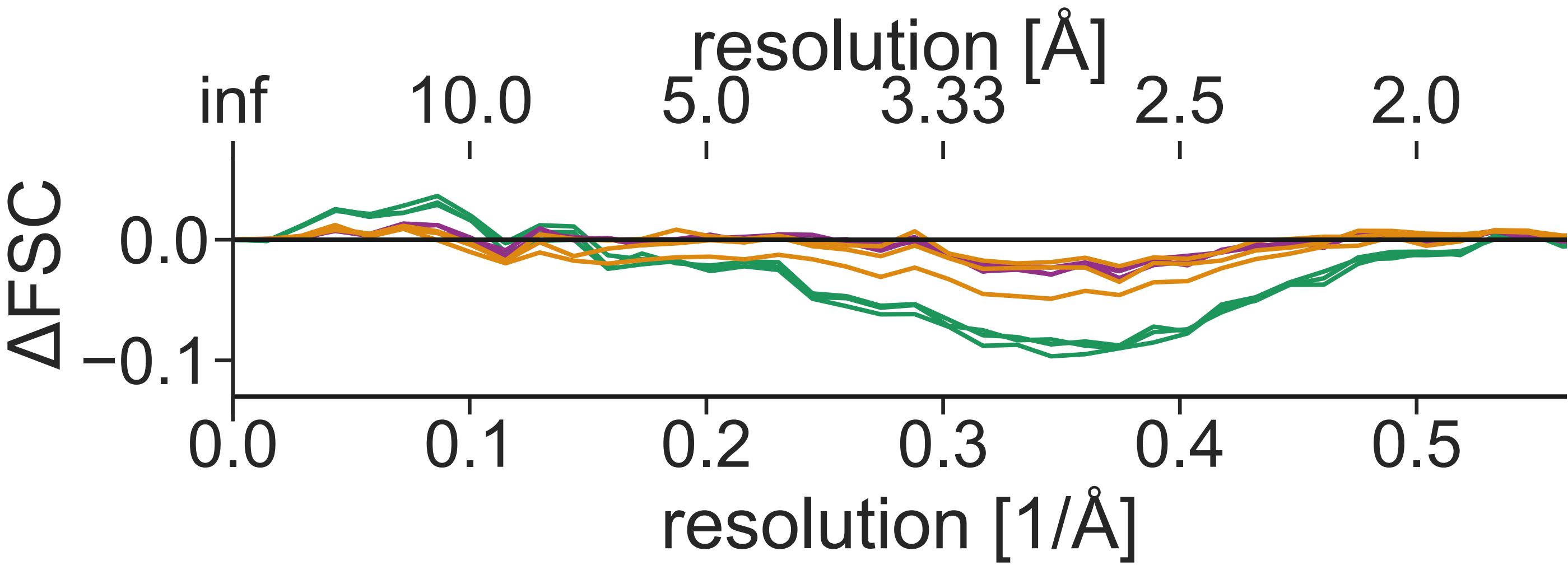

Supplement: S7 Fig — Difference in FSC to manually built model for inner-product (purple), cross-correlation (ochre), relative-entropy swapped (dark blue) and relative-entropy (green) based refinements with best accepted FSC (dotted) and final FSC (solid). (PDF) [file pcbi.1011255.s015.pdf]
