# Supplementary material for: Gentle and fast all-atom model refinement to cryo-EM densities via a maximum likelihood approach
Source: PLoS Comput Biol. 2023 Jul 31;19(7):e1011255. doi: 10.1371/journal.pcbi.1011255 (PMC10427019; doi:10.1371/journal.pcbi.1011255)
Supplement: Attachment — Submitted filename: review.pdf [file pcbi.1011255.s016.pdf]

Summary

The paper outlines an MD-based approach to fit atomic structures into cryo-EM maps. The authors extend GROMACS with a new density fitting score based on the relative entropy / KL-divergence. To balance the relative entropy against the force field, an adaptive force scaling approach is introduced. The approach is illustrated for a simulated map of a helix and an experimental map of rabbit-muscle-aldolase. The authors find a superior performance of their score in comparison to more traditional scores such as the cross-correlation coefficient (CCC) at least for these low-noise targets.

It is nice to see state-of-the-art all-atom modeling being used to combine cryo-EM maps with a physically realistic force field. Moreover, the automated force balancing approach is an interesting way of combining experimental data with a force field. However, given the conceptual difficulties in the derivation of the relative entropy score and the limited tests carried out by the authors, I currently don't see why this work merits a publication in PLoS Computational Biology.

Major points

Poisson model

The relative entropy score is derived from a Poisson likelihood. However, the Poisson distribution is defined for count data (nonnegative integers). Therefore, the Poisson distribution can't be a likelihood for the density map ρ (an array of nonnegative floats) without a conversion of the experimental voxel values ρ_v to counts. The scaling factor r (introduced on page 8) could achieve this conversion, but you would still need a floor operation or something similar to obtain counts from $r\rho_v$. However, you seem to set r such that the experimental map ρ normalizes to one. Therefore, a more appropriate model for ρ might be a Dirichlet distribution resulting in a score $k \sum_v \rho_v^s \log \rho_v$, which is somewhat related to the KL divergence but swaps the role of ρ and ρ^s (here the "force constant" k could be introduced quite naturally via the Dirichlet model).

You should at least comment on these assumptions/choices, because it also affects the treatment of the "force constant": The scaling factor r would play the role of a force constant, and it should also be estimated by Bayesian means.

Derivation of the relative entropy potential

I have a problem with your equation (1) in the Supporting Information and the introduction of the force constant k. If you start with a probabilistic model for the EM map $p(\rho|\rho^s)$, then $-\log p(\rho|\rho^s)$ is already the contribution to the overall "energy" stemming from the data.

There is no room for arbitrary scaling with a force constant k (which would mean that you raise $p(\rho|\rho^s)$ to powers of k), since $p(\rho|\rho^s)$ needs to be normalized wrt ρ. So either k is known and fixed, or the additional constant c depends on k, and it's only if this k-dependence is properly taken into account, that k can be estimated.

If you want to introduce a parameter k that behaves like a force constant, it should come from the probabilistic model $p(\rho|\rho^s)$ (in which case k should also occur right of the conditioning stroke in $p(\rho|\rho^s)$). For example, in case of a Gaussian likelihood $p(\rho|\rho^s, k) = \prod_v N(\rho_v; \rho_v^s, 1/k)$ where $N(x; \mu, \sigma^2)$ is a Normal distribution (for variable x) with mean μ and variance σ^2, the inverse variance $k = 1/\sigma^2$ behaves like a force constant and can be estimated using Bayesian inference.

Adaptive force scaling

The adaptive force scaling approach seems to be purely heuristic. Can't you use probabilistic reasoning to estimate the force constant? On page 10, you state: "The force constant for density-guided simulations cannot be derived from the cryo-EM density alone and thus needs to be set heuristically." - Why? Bayes should allow you to estimate the force constant. As I explained in my last comment, I think that the defect is due to the inappropriate likelihood. Or stated differently: In a truly Bayesian approach, there is no room for "force balancing" or similar heuristics.

Bayesian approach

The authors derive their score by adopting a probabilistic argument (with the caveat explained in my first comment). The Poisson distribution is used to model density larger than an offset in a voxel-wise independent fashion. Other than that the authors don't take advantage of the Bayesian machinery, which should allow them to estimate the force constant. Instead they come up with their own heuristic (adaptive force balancing) of tuning the force constant.

In principle, I think that labels don't matter really. So whether you call your approach Bayesian or something else is not of much importance (e.g. you could also call it regularized maximum likelihood). However, in my opinion you are missing a chance here in that you don't take full advantage of the Bayesian inference machinery in your treatment.

Tests

It is hard to judge the performance of your approach, if it is only tested on a single experimental map, and not compared with other refinement approaches. Therefore, I think more tests with different systems and data as well as a comparison with other refinement approaches are needed before your work can be published.

Tests with aldolase data

I have some questions regarding your refinement against the aldolase map. It would be useful to clarify these in your reply and perhaps also in the manuscript:

- The aldolase map shows a four-fold symmetry. Do you use symmetry restraints during the refinement?
- How do you balance the forces resulting from the other similarity scores (inner product, cross correlation)? Also by using the adaptive force balancing heuristic?
- Which offset/threshold did you use in your simulations? Is it the recommended contour level?
- You argue and illustrate that the density fitting score has favorable properties (e.g. in Fig. 2). However, in terms of quality measures such as Ramachandran statistics the structures obtained with relative entropy potential seem to be slightly worse than the structures fitted with the other similarity scores.
- What are the final values for the force constant? Perhaps you could also plot traces showing the evolution of the force constant during refinement.
- The reported resolution of the map EMD-21023 is 2.13 Å. Why do you report average FSC values at 2.17 Å resolution in Fig. 5c?

Minor points

- Figure 1: I find it a bit weird that the experimental map (in blue) and theoretical map (in red) appear to represent a largely different volume. Are they contoured differently? Also in Fig. 4, the straight helix seems to be the model and the bent helix the density map, whereas in Fig. 1 it's the other way around.
- Figure 3: I appreciate your effort in explaining the adaptive force scaling in Figure 3. However, I found it difficult to understand how adaptive force balancing works by looking at the figure. I had to read the caption and text to understand it. The explanation in subsection C of the Methods section is much clearer and more useful. Either revise figure 3 or remove it.
- Page 8: "which can seen"
- Page 19: "The newly defined potential has favourable features like long-rangedness and low ruggedness" could be phrased more elegantly.
- Page 19: "which avoids false local minima during refinement" - there are no *false* local minima.

- Page 1, Supporting Info: There seem to be two uses of the symbol k. In equation (1), k is the force constant. In the explanation between equations (3) and (4) ("$k = \rho_v$"), k seems to be a count (since you are talking about the Poisson distribution). Please clarify and perhaps use different symbols.
