# Supplementary material for: Gentle and fast all-atom model refinement to cryo-EM densities via a maximum likelihood approach
Source: PLoS Comput Biol. 2023 Jul 31;19(7):e1011255. doi: 10.1371/journal.pcbi.1011255 (PMC10427019; doi:10.1371/journal.pcbi.1011255)
Supplement: Attachment — Submitted filename: blau_plos_response.pdf [file pcbi.1011255.s017.pdf]

Stockholm University

Stockholm February 27, 2023

Response to reviewer comments - PCOMPBIOL-D-22-01441

Dear Prof. Hub,

Thank you very much for the highly useful reviewer feedback, and also for allocating some extra time to properly address the comments as Dr. Blau is currently working full time in industry and had to do the work in evenings and weekends. The suggestions were good, and we have incorporated virtually all of them in the new version of the manuscript - including a fourth new refinement potential due to the ideas of reviewer 3! For your convenience, we have created a table-of-contents for the specific review comments organized into subsections, with reviewer text in black and our response in blue italics.

1 Reviewer 1 **2**
- 1.1 Test different poses noise-free helix 2
- 1.2 Test different scores 2
- 1.3 Show relative entropy is good also for other biological structures 2

2 Reviewer 2 **2**
- 2.1 Clarify that potential energy landscape ruggedness and density ruggedness are not the same thing 3
- 2.2 Motivate the three sigma potential energy threshold 3
- 2.3 Motivate use of half-maps for fitting 3
- 2.4 Expand on the impossibility to estimate certain model parameters 4
- 2.5 Address relative-entropy performance when the model misses parts 4
- 2.6 Edit manuscript to remove "electron density" map term 4
- 2.7 Spreading on grid - remove noise assumption 4
- 2.8 Motivate choice of unit scattering amplitude 5
- 2.9 Typos 5

3 Reviewer 3 **5**
- 3.1 Poisson model 5
- 3.2 Derivation of the relative entropy potential 6
- 3.3 Adaptive force scaling 6
- 3.4 Bayesian approach 6
- 3.5 Tests 7
- 3.6 Tests with aldolase data 7
- 3.7 Minor points 8

1 Reviewer 1

In this work, the authors proposed to use relative entropy derived from Bayes' approach in all-atom model refinement to cryo-EM density data. In the model refinement, molecular force field and similarity bias force are both used. The conventional similarity bias forces are inner product, cross correlation, and so on. The proposed relative entropy seems to have advantages over these conventional similarity bias forces because the effect of similarity bias is longer range even though the overlap between structural models and cryo-EM densities is very small. Adaptive force scaling looks also useful for adjusting the good balance between molecular force field and similarity bias forces. In general, this work can provide new insight and useful information in the all-atom model refinement to cryo-EM density data. We would like to suggest several points to improve the manuscript and to clarify the advantages of relative entropy in the refinement.

Appreciated, thanks!

1.1 Test different poses noise-free helix

(1) For noise-free data, is it possible to test different initial structures? For instance, not only completely unaligned structures but also (one or two residue) miss-aligned ones could be tested.

This is a good idea. Although we don't have room for an exhaustive assessment of noise-free data (since we also wanted to include another real example system, as well as the new refinement potential), we have now updated Figure 3 to show both starting points for the helix refinement, better indicating the quite aligned starting structure and the misaligned (no overlap) starting pose.

1.2 Test different scores

(2) To compare the performance of inner-product, cross-correlation with relative-entropy, not only FSC but also EMRinger, MolProbity, CABLAM should be shown.

Check. We have now included all these scores both for aldolase and the new (second) biological system groel in the supplementary material.

1.3 Show relative entropy is good also for other biological structures

(3) In Figure 6, they used distorted models for showing the performance of relative-entropy approach. We suggest them to show more practical examples for showing the performance. For instance, there are several proteins whose structures were determined with X-ray crystallography and another structures were solved with cryo-EM at the lower resolution. We would like to see how the relative entropy shows good performance to refine all-atom models with lower resolution cryo-EM densities using the X-ray structure at the different physiological states. As we suggested, not only FSC but also EMRinger, MolProbity, and CABLAM could be used to investigate the performance.

We have now added a refinement of a GroEl subunit between two previously determined states and show the results in a new Figure 6 which has proven useful to show some strengths - and also potential challenges - for our newly defined refinement potentials. Structure scores are added to the supplementary material.

2 Reviewer 2

The manuscript by Blau et al. describes an algorithm to refine protein structures against cryo-EM density maps that is based on molecular dynamics simulations. In their description, the assumption is made, that the EM density map describes a single conformation and the goal of the refinement is a single, best-fitting model.

Applications to simulated and experimental density maps are shown. Overall, this is a very interesting manuscript, which describes a new development of the MD-based structure refinement in Gromacs. The method seems to be well applicable and fast, it is therefore a valuable contribution for the (computational) structural biology community and well suited for PLOS Computational Biology.

Thanks for the kind comments!

Here are a few points that need to be clarified:

2.1 Clarify that potential energy landscape ruggedness and density ruggedness are not the same thing

- A new refinement potential is derived, which results in the Kullback-Leibler divergence between model density and EM density. The authors emphasize that this refinement potential is smooth. I would think that ideally, all experimental information should be used during the refinement. I understand, that high-resolution density maps are rugged and lead to slower convergence, since many barriers need to be crossed, but this is often accounted by a stepwise refinement, starting with low-pass filtered maps (as e.g. described in cascade MDFF), and then at later stages of the refinement, the full-resolution maps are used.

Indeed, we fully agree on this. Our newly defined potential is fully orthogonal to resolution-based approached to smoothen the energy landscape and should work very well in combination with these (which we intend to pursue in the future). We now acknowledge this fact in the discussion.

It does not sound like a desirable feature to use only a smooth refinement potential throughout and thereby possibly ignoring valuable high-resolution information. The authors might want to clarify, whether indeed high-resolution is potentially ignored with their approach.

This is a very good comment! The relative entropy based potential does not forego any information in the density map (i.e. "smoothening" in terms of averaging), but rather achieves a smooth potential by avoiding to add (rough) features to the potential energy landscape that have no actual correspondence to data in the map. We have now updated Figure 2 to make this point clearer and point out the "correct" solution in the potential energy landscape.

2.2 Motivate the three sigma potential energy threshold

- Where does the 3sigma potential energy threshold come from? This is not really described in the text.

To be honest, this was merely an ad-hoc value we used for convenience, but since it does not provide any real useful value, we have now simply removed it (in particular since we also wanted to make room for the new material added in response to reviewer comments) - it is better to compare the full time evolution of the FSC fits as we do in Fig 5c.

2.3 Motivate use of half-maps for fitting

- In the applications to experimental data, the model was fit against half map1? Why was that done? The half maps are usually the raw output of the density refinement program (e.g. Relion), the maps are typically not filtered and not sharpened and should therefore not be used for structure refinement.

Thanks for pointing out this inconsistency. We have now use the full map for the refinement instead. Having said that, fitting to half-maps may provide a means to analyze over-fitting in the data, but due to limited space we decided not to touch this topic in the current manuscript.

- The FSC threshold for the resolution definition is 0.143 when comparing the two half maps (after a gold-standard refinement). The FSC threshold for comparing a model map with the full EM map (sum of both half maps) (also referred to as the model-map cross-resolution) is 0.5.
That means, if the half map FSC is 0.143 at a resolution value XÅ, the model-map FSC should have a value of 0.5 at the same resolution XÅ. However, in figure 5 and 6 this is not the case.
Here, the model is compared to only a half map, which is unusual and makes this comparison more difficult. This needs to be clarified.

Point taken. As described above, we have now updated all figures to show refinement to full maps.

2.4 Expand on the impossibility to estimate certain model parameters

- page 10: "The force constant for density-guided simulations cannot be derived from the cryo-EM density alone and thus needs to be set heuristically."
In principle, the force constant could in fact be determined from the cryo-EM data, it requires to model the error of the density map. The main motivation to use Bayes (in my opinion) is that it does not require any tunable parameters if all errors are modeled. However, in this work, there are still several tunable parameters: the force-constant, the filter of the density maps, and the 3sigma potential cut-off selects the best structure from the trajectory. The error of the density map could in principle be modeled based on the FSC. When I read "Bayes" in the title, I immediately expected that the force constant would be obtained by modeling the experimental errors. It might be helpful to the reader to clarify, why this was not done.

This is a fair point. Long-term we would like to get there, but in practice there are challenges related to models both for the experimental and force field errors. We have added a section in the derivation of the potential to explain why we determined the force constant as we did and appreciate that in many cases, we would like to achieve a constraining potential that produces an ensemble matching the true variance in the data and outline a way to achieve this in the discussion on local resolution measures.

2.5 Address relative-entropy performance when the model misses parts

- The relative-entropy based density potential enforces a global agreement of model and density. How does that affect the typical practical problem of missing model (e.g. missing long loops, missing domains)? Would you expect larger distortions than with the traditional refinement target functions?

Indeed - it is correct that proper automatic treatment of missing data is a shortcoming of the potential that is inherently missing in the current model. For now we adress this (when relevant) by e.g. excluding loops from refinement. We now acknowledge this both in the overall discussion and specifically in the discussion of the GroEl simulation results.

2.6 Edit manuscript to remove "electron density" map term

- Throughout the manuscript: Cryo-EM does not yield an "electron density" map, but an electrostatic potential map. Please do not use "electron density". (X-ray diffraction yields electron density)

Guilty as charged. It escaped our attention that we kept using the term and rather prefer to speak just of "cryo-EM density", or, with model assumptions, of an "electron interaction density".

2.7 Spreading on grid - remove noise assumption

Appendix S1, "II Gaussian Spreading on a Grid":
"we assume Gaussian spatial noise", I do not understand why this has anything to do with noise? This is just a convolution with a Gaussian, or not?

Yes, in effect this is what is happening. Sorry for the confusion; we have now simplified the paragraph to make this clearer.

2.8 Motivate choice of unit scattering amplitude

And how is the factor A chosen, this should depend on atom type. There is a reference to a textbook by Kirkland, but I think it would be helpful to just list values for the few most relevant atom types in proteins.

Good point. We now list the factors in the supplementary material and have updated the script to reflect the fact that we approximate these factors with unity for all heavy atoms - an approximation due to implementation details which we found works well in our case.

2.9 Typos

page 7: "vitr*e*ous ice"
page 10: "This issue can *be* alleviated in..."
page 14: "excees" -> "exceeds"
Appendix page 4: "Wi*gn*er-Seitz"

Apologies; thanks for spotting these - they are now all fixed.

3 Reviewer 3

The paper outlines an MD-based approach to fit atomic structures into cryo-EM maps. The authors extend GROMACS with a new density fitting score based on the relative entropy / KL-divergence. To balance the relative entropy against the force field, an adaptive force scaling approach is introduced. The approach is illustrated for a simulated map of a helix and an experimental map of rabbit-muscle-aldolase. The authors find a superior performance of their score in comparison to more traditional scores such as the cross-correlation coefficient(CCC) at least for these low-noise targets. It is nice to see state-of-the-art all-atom modeling being used to combine cryo-EM maps with a physically realistic force field. Moreover, the automated force balancing approach is an interesting way of combining experimental data with a force field. However, given the conceptual difficulties in the derivation of the relative entropy score and the limited tests carried out by the authors, I currently don't see why this work merits a publication in PLoS Computational Biology.

Thank you; we really appreciate this reviewers careful review of the equation derivations (which has resulted in a new fourth refinement potential!), and have tried to incorporate all suggestions to the extent possible without completely redesigning the fitting, as well as added more test cases. In particular, the suggestions of this reviewed led us to implement a new fourth type of refinement potential (swapped-relative-entropy) that behaves slightly differently (for better or worse). We have expanded the derivation part in the main text to cover the two different views/assumptions of the scattering probability that lead to the different potentials.

3.1 Poisson model

The relative entropy score is derived from a Poisson likelihood. However, the Poisson distribution is defined for count data (nonnegative integers). Therefore, the Poisson distribution can't be a likelihood for the density map ρ (an array of nonnegative floats) without a conversion of the experimental voxel values ρ_v to counts. The scaling factor r (introduced on page 8) could achieve this conversion, but you would still need a floor operation or something similar to obtain counts from ρ_v.

Good point. We have now added some sentences to clarify the rescaling of density values, omission of negative density values, and further explain our use of vitreous ice as zero line. We have also expanded on our model assumptions, focusing more on minimal model assumptions that for now

yield a beneficial potential that is implementable within an molecular dynamics framework than as a complete as possible model - because the reviewer is quite right there are limitations to it.

However, you seem to set r such that the experimental map ρ normalizes to one. Therefore, a more appropriate model for ρ might be a Dirichlet distribution resulting in a score which is somewhat related to the KL divergence but swaps the role of (here the "force constant" k could be introduced quite naturally via the Dirichlet model). You should at least comment on these assumptions/choices, because it also affects the treatment of the "force constant": The scaling factor would play the role of a force constant, and it should also be estimated by Bayesian means.

Thank you so much for this suggestion! We have implemented this as a new - fourth - potential (which we call "swapped-relative-entropy", describe it in the manuscript, and find that it provides a practical and nice bridge between the inner-product and our Poisson statistics based potential.

3.2 Derivation of the relative entropy potential

I have a problem with your equation (1) in the Supporting Information and the introduction of the force constant k. If you start with a probabilistic model for the EM map $p(\rho|\rho^s)$, then log $p(\rho|\rho^s)$ is already the contribution to the overall "energy" stemming from the data. There is no room for arbitrary scaling with a force constant k (which would mean that you raise $p(\rho|\rho^s)$ to powers of k), since $p(\rho|\rho^s)$ needs to be normalized wrt k So either k is known and fixed, or the additional constant c depends on k, and it's only if this k-dependence is properly taken into account, that k can be estimated. If you want to introduce a parameter k that behaves like a force constant, it should come from the probabilistic model $p(\rho|\rho^s)$ (in which case k should also occur right of the conditioning stroke in $p(\rho|\rho^s)$). For example, in case of a Gaussian likelihood $p(\rho|\rho^s,k) = \Pi_v N(\rho_v;\rho_v^s,1/k)$ where $N(x;\mu,\sigma^2)$ is a Normal distribution (for variable x) with mean μ and variance σ^2, the inverse variance $k = 1/\sigma^2$ behaves like a force constant and can be estimated using Bayesian inference.

Very fair point. We have now introduced the scaling constant in our model derivation and improved the way where we show how we link this to the force constant.

3.3 Adaptive force scaling

The adaptive force scaling approach seems to be purely heuristic. Can't you use probabilistic reasoning to estimate the force constant? On page 10, you state: "The force constant for density-guided simulations cannot be derived from the cryo-EM density alone and thus needs to be set heuristically." - Why? Bayes should allow you to estimate the force constant. As I explained in my last comment, I think that the defect is due to the inappropriate likelihood. Or stated differently: In a truly Bayesian approach, there is no room for "force balancing" or similar heuristics.

This is a good point, which is formally correct - and we have adjusted the phrasing to clarify that it is merely a practical, rather than theoretical, limitation. However, in practice we are both limited by the complex density reconstruction algorithms which means the prior distribution for the parameter is not known, and in practical simulations we also have the challenge that too large force constants would lead to crashes, so we anyway need to limit it heuristically. However, we have now added a section in "Bayes approaches yield negative..." to describe in more detail why we did not determine the force constant automatically - but it is worth pursuing in the future.

3.4 Bayesian approach

The authors derive their score by adopting a probabilistic argument (with the caveat explained in my first comment). The Poisson distribution is used to model density larger than an offset in a voxel-wise independent fashion. Other than that the authors don't take advantage of the Bayesian machinery, which should allow them to estimate the force constant. Instead they come up with their own heuristic

(adaptive force balancing) of tuning the force constant. In principle, I think that labels don't matter really. So whether you call your approach Bayesian or something else is not of much importance (e.g. you could also call it regularized maximum likelihood). However, in my opinion you are missing a chance here in that you don't take full advantage of the Bayesian inference machinery in your treatment.

See previous item. We definitely agree that a fully and correctly defined force with no room for pure heuristics would be favourable for eg. correctly defining ensembles of structure that "fit" a density. However, for the purpose of finding the most likely single structure in combination with molecular dynamics we also presently face practical limitations, and at least in a first version we believe the adaptive force scaling is useful in the sense of a heuristic search - but as we now comment in the text, in a full Bayesian approach the scaling factor should ideally be possible to integrate out.

3.5 Tests

It is hard to judge the performance of your approach, if it is only tested on a single experimental map, and not compared with other refinement approaches. Therefore, I think more tests with different systems and data as well as a comparison with other refinement approaches are needed before your work can be published.

Point taken. In addition to comparing the three refinement approaches commonly used today for the noise-free helix case as well as aldolase, we have now also added another experimental system, a GroEl subunit. This was helpful in highlighting further benefits and challenges to our approach. Especially the inclusion of missing model density poses a challenge to our approach that will have to be adressed by further research, while we observe, on the other hand, excellent fits using the adaptive force-scaling model.

3.6 Tests with aldolase data

I have some questions regarding your refinement against the aldolase map. It would be useful to clarify these in your reply and perhaps also in the manuscript:

- The aldolase map shows a four-fold symmetry. Do you use symmetry restraints during the refinement?
  No, we did not use symmetry restrains, mainly because real molecules in solution are not subject to such restraints. This is now clarified in the methods section.
- How do you balance the forces resulting from the other similarity scores (inner product, cross correlation)? Also by using the adaptive force balancing heuristic?
  Yes, we used adaptive force-scaling throughout and found this was one of the necessary ingredients to make the potentials comparable. We have now clarified this in the methods section.
- Which offset/threshold did you use in your simulations? Is it the recommended contour level?
  We did not contour any density data but used the full density information. Note however, that, per definition, we exempt negative density voxel values from the calculation of the relative entropy potential.
- You argue and illustrate that the density fitting score has favorable properties (e.g. in Fig. 2). However, in terms of quality measures such as Ramachandran statistics the structures obtained with relative entropy potential seem to be slightly worse than the structures fitted with the other similarity scores.
  This is a good point, which ultimately comes down to how much we want to emphasize force field vs. fit-do-density vs. quality measures (which we now include more of). We agree that the model statistics for the refinement runs are good, but not perfect and may benefit from further treatment, eg., a short energy minimization without any restraints to fix stereogeometry and would expect model statistics differences to become smaller again. However, rather than doing it ad hoc for a

single structure or two, we believe it is better to show the raw results and leave systematic (potentially iterative) refinement pipeline design for a future large-scale study. For this reason (not to mention space concerns), we chose not to pursue this avenue in the current publication.

- What are the final values for the force constant? Perhaps you could also plot traces showing the evolution of the force constant during refinement.
  We have now added SI figure 10 to illustrate this.
- The reported resolution of the map EMD-21023 is 2.13 Å. Why do you report average FSC values at 2.17 Å resolution in Fig. 5c?
  Thanks for noticing! This fence-post error when binning the fourier correlation shells has now been corrected.

3.7 Minor points

- Figure 1: I find it a bit weird that the experimental map (in blue) and theoretical map (in red) appear to represent a largely different volume. Are they contoured differently?
  We tried to reflect in Figure 1 that the simulated density need not be as close as possible to the experimental density in resolution, as argued for in "Deriving an optimal model density generation for cryo-EM data refinement."
- Also in Fig. 4, the straight helix seems to be the model and the bent helix the density map, whereas in Fig. 1 it's the other way around.
  Fair point; in hindsight this might not have been ideal, but we have made sure we use completely different color schemes and representations, and explicit labels, so hopefully they will not be confused.
- Figure 3: I appreciate your effort in explaining the adaptive force scaling in Figure 3. However, I found it difficult to understand how adaptive force balancing works by looking at the figure. I had to read the caption and text to understand it. The explanation in subsection C of the Methods section is much clearer and more useful. Either revise figure 3 or remove it.
  Appreciated; figure 3 has now been revised. We hope that it now shows the adaptive force scaling more clearly, especially in panel B.
- Page 8: "which can seen"
  Thanks, fixed.
- Page 19: "The newly defined potential has favourable features like long-rangedness and low ruggedness" could be phrased more elegantly.
  We made an attempt to reformulate our conclusions here to both be more easily understood and more concrete.
- Page 19: "which avoids false local minima during refinement" - there are no false local minima.
  Point taken. We have reformulated this and hope it is clearer what we mean.
- Page 1, Supporting Info: There seem to be two uses of the symbol k. In equation (1), k is the force constant. In the explanation between equations (3) and (4) ("$k=\rho_V$"), k seems to be a count (since you are talking about the Poisson distribution). Please clarify and perhaps use different symbols.
  Check. We have updated the supporting information to be clearer on this aspect.

Once again, thank you so much for a number of very valuable and productive review comments that have helped improve the manuscript.

Sincerely,

/Erik Lindahl/
