# Supplementary material for: Gentle and fast all-atom model refinement to cryo-EM densities via a maximum likelihood approach
Source: PLoS Comput Biol. 2023 Jul 31;19(7):e1011255. doi: 10.1371/journal.pcbi.1011255 (PMC10427019; doi:10.1371/journal.pcbi.1011255)
Supplement: Attachment — Submitted filename: review2.pdf [file pcbi.1011255.s018.pdf]

Summary

I appreciate the authors' efforts to improve the manuscript. However, the main issues that I have with the formal derivations and the fact that the paper falls short of applying Bayesian techniques to estimate force constant-like parameters remain unresolved. Therefore, I don't have much to add and stand by my previous review for the most part. However, let me try to clarify some points (at the risk of being lengthy).

Major points

Force constant k

I am still not convinced by the derivation of the total potential energy U_{tot}, combining score S with a force field U_{ff}. Equations (1) and (4) state

$$U_{\text{tot}}(\vec{x}, \rho) = U_{\text{ff}}(\vec{x}) - kS(\rho, \vec{x}), \qquad \frac{k}{k_B T} S(\rho, \vec{x}) = \log p(\rho|\vec{x})$$

whereby the force constant k sneaks into the framework. Later on you propose to tune k with adaptive force scaling. But why is there a force constant k in the first place? One can rewrite

$$U_{\text{tot}}(\vec{x}, \rho) = U_{\text{ff}}(\vec{x}) - k_B T \log p(\rho|\vec{x})\,.$$

The force constant k disappears, and there is nothing that needs to be tuned. For example, upon plugging in Eq. (8) from Appendix S1 the total energy of the Poisson model becomes

$$U_{\text{tot}}(\vec{x}, \rho) = U_{\text{ff}}(\vec{x}) - k_B T \sum_v \rho_v \log(\rho_v^s(\vec{x})/\rho_v)\,.$$

Therefore, in a Bayesian approach there is no need for force scaling because there is no k, unless a k-like parameters enters the model via $p(\rho|\vec{x})$ (in which case k can be estimated via its conditional posterior).

Since k is not derived from $p(\rho|\vec{x})$, one might consider it an algorithmic parameter such as an inverse temperature in annealing. But then k should approach 1 (because eventually we aim to simulate the correct posterior and not a model in which the weight of the data score is too small/high). Figure S3 shows that k exceeds 1 by many orders of magnitude. Therefore, k cannot merely be seen as an inverse temperature that helps us find the global optimum.

I also find Fig. S3 concerning in that k fails to converge to a stable value. This risks overfitting, because the data acquire an increasingly large weight. Figure S3 suggests that the length of the refinement simulations varies with the score. What is the criterion to stop the simulation? In particular, in case of the entropy related scores it seems that the force constant would dramatically increase if the simulation ran longer.

Dirichlet model

It's nice to see that the Dirichlet model seems to be useful for EM fitting. But your use of the Dirichlet distribution misses the chance to introduce a k-like parameter, the scaling factor s, probabilistically and estimate it with Bayesian means. The full likelihood resulting from the Dirichlet distribution is

$$p(\rho|\vec{x}, s) = \frac{\Gamma(s \sum_v \rho_v^s(\vec{x}))}{\prod_v \Gamma(s\rho_v^s(\vec{x}))} \prod_v \rho_v^{s\rho_v^s(\vec{x})-1}$$

where Γ is the Gamma function and $\sum_v \rho_v = 1$ (your Eq. (5) is only a proportionality). The resulting score is

$$\log p(\rho|\vec{x}, s) = s \sum_v \rho_v^s(\vec{x}) \log \rho_v + c(\vec{x}, s)$$

where c depends on both $\vec{x}$ and s:

$$c(\vec{x}, s) = \log \Gamma\big(s \sum_v \rho_v^s(\vec{x})\big) - \sum_v \log \Gamma(s\rho_v^s(\vec{x}))\,.$$

Your score S_I (Eq. 6) omits c which assumes that c varies only weakly with $\vec{x}$. You should justify why this approximation holds (one can argue that $\sum_v \rho_v^s(\vec{x})$ is approximately constant, but then there is still the second sum in c).

At the bottom of page 8, you state that s is "a free scaling paramter". Why don't you estimate s based on its conditional posterior

$$p(s|\vec{x}, \rho) \propto s^{-1} \frac{\Gamma(s \sum_v \rho_v^s(\vec{x}))}{\prod_v \Gamma(s\rho_v^s(\vec{x}))} \exp\Big\{ s \sum_v \rho_v^s(\vec{x}) \log \rho_v \Big\}$$

assuming, for example, a Jeffreys prior s^{-1}? It remains unclear to me, why you don't use Bayesian techniques such as sampling or maximization of $p(s|\vec{x}, \rho)$ to estimate s.

Poisson model

Similar arguments apply to the Poisson model:

$$\log p(\rho|\vec{x}, r, N) = r \sum_v \rho_v \log\left(N\rho_v^s(\vec{x})\right) - N \sum_v \rho_v^s(\vec{x}) - \sum_v \log[(r\rho_v)!]$$

with scaling factor r and number of incident electrons N. Again, one could argue that $\sum_v \rho_v^s(\vec{x})$ is constant such that the $\vec{x}$-dependent score is indeed $r \sum_v \rho_v \log \rho_v^s(\vec{x})$. Therefore, r acts like a "force constant" with posterior

$$p(r|\vec{x}, \rho, N) \propto \exp\left\{ r \sum_v \rho_v \log\big(N\rho_v^s(\vec{x})\big) - \sum_v \log \Gamma(r\rho_v + 1) \right\}$$

where the factorial $(r\rho_v)!$ is replaced by $\Gamma(r\rho_v+1)$. As in the case of the Dirichlet model, you could sample or maximize $p(r|\vec{x}, \rho, N)$ to estimate r which would require knowledge of N.

On page 9 you acknowledge that r could be integrated out, but state that "*the prior distribution for this parameter is unknown and hard to estimate correctly due to the complex density reconstruction algorithm. Even if we were to determine this scaling correctly, a "correct" force scale in such a Bayesian setting would most likely result in very large forces for conformations with significant deviants at the start of simulations, resulting in numerical instabilities in a molecular dynamics setting.*" First, the prior distribution $p(r)$ is neither unknown or known, but simply *assigned* by you. Second, it's specific form won't play an important role because the data term will dominate. Therefore, you could pick any prior that is conventionally used for scale parameters such as Jeffreys' prior or a Gamma distribution. Third, I don't understand why an estimated scaling factor "should result in very large forces" at the start of the simulation. Typically, structures that do not fit the data well will result in small estimated force constants.

Shortcomings of formal derivations

Overall, I find parts of your formal arguments unsatisfactory. I understand that PLoS Comp Biol is not a mathematics or physics journal, but the equations that you present should be correct and have a clear justification.

As an example, consider the treatment of scaling factor r for the experimental map ρ. You treat r as a nuisance parameter by defining a prior $p(r)$ and write the likelihood of ρ as a mixture over the scales. This mixture should read:

$$\int_0^\infty p(r)\, p(\rho|\vec{x}, r)\, dr\,.$$

Your equation on top of page 8 lacks the differential dr and has a wrongly positioned upper limit. You never introduce the prior $p(r)$ over the scale r other than implicitly via this equation. Comments on suitable choices for $p(r)$ are missing (it's only later on page 9 that you mention $p(r)$ as being an "unknown prior distribution").

On page 9, in Eq. (7) you then introduce

$$\frac{k}{k_B T} S_{II}(\rho, \rho^s) = \int_0^\infty p(r) \sum_v r\rho_v \log\left(\rho_v^s(\vec{x})/r\rho_v\right)\, dr\,.$$

Again, your expression has a problem since the parentheses should be put around the argument of the logarithm. A more severe shortcoming is that the rationale behind Eq. (7) remains unclear. It does not follow from the scale mixture $\int_0^\infty p(r)\, p(\rho|\vec{x}, r)\, dr$ and the Poisson likelihood:

$$p(\rho|\vec{x}, r, N) = \prod_v \frac{[N\rho_v^s(\vec{x})]^{r\rho_v}}{(r\rho_v)!} e^{-N\rho_v^s(\vec{x})}\,.$$

Instead, Eq. (7) looks like "$\int_0^\infty p(r) \log p(\rho|\vec{x}, r)\, dr$" for the special case of a Poisson likelihood, which is different from "$\log \left(\int_0^\infty p(r)\, p(\rho|\vec{x}, r)\, dr\right)$" (however, both expressions are related via Jensen's inequality). Your score $S_{II}(\rho, \rho^s)$ could be transformed to

$$\overline{r} \sum_v \rho_v \log \left(\rho_v^s(\vec{x})/\rho_v\right) - \overline{r \log r} \sum_v \rho_v$$

where $\overline{f(r)} = \int_0^\infty f(r)\, p(r)\, dr$, but again the main problem is that you don't provide any justification for the expression itself.

Performance of the entropic scores

In addition to the problems with the formal arguments, I don't see how (as stated in the Abstract) "*the Bayesian refinement potential outperforms alternatives, and the adaptive force scaling appears to also aid existing refinement potentials*" based on your results. For example, tables S8 and S9 shows that the conventional scores (cross-correlation coefficient and inner product) produce structures which are both very accurate (low RMSD to published structure) and show good model statistics. Similar observations can be made for the new simulations with data on GroEL.

Comparison with other methods

A comparison with other refinement methods using the same data is still missing.

Minor points:

- In your simulations, which value do you use for s (scaling factor of the Dirichlet model)? Or is s identified with k and treated with force scaling?
- Page 8: Striktly speaking, Eq. (5) is no equality, but only a proportionality.
- The left parenthesis "(" in Eqs. (7) and (8) should be right of "log" (rather than left).
