# Supplementary material for: Gentle and fast all-atom model refinement to cryo-EM densities via a maximum likelihood approach
Source: PLoS Comput Biol. 2023 Jul 31;19(7):e1011255. doi: 10.1371/journal.pcbi.1011255 (PMC10427019; doi:10.1371/journal.pcbi.1011255)
Supplement: Attachment — Submitted filename: response_to_reviewers.pdf [file pcbi.1011255.s019.pdf]

Stockholm May 25, 2023

Response to reviewer comments - PCOMPBIOL-D-22-01441

Dear Prof. Hub,

Thank you for the very detailed comments and efforts from the third reviewer (as well as you and the other reviewers, of course) - we deeply appreciate it, and the only reason it took is some time to get back is due to Dr. Blau's limited availability. We hope we have adressed the remaining comments - or at least made it explicitly clear in discussions and results what the limitations are.

Editor

Reviewers 1 and 2 confirmed that their comments have been largely addressed. Reviewer 3, however, points out that several mathematical equations as well as new text added during the revision are not justified or not sufficiently explained. His criticism on the fact that the method is not "Bayesian" remains (because it is never sampled from the posterior). From Reviewer 3's report and from my own reading, there is no need for new simulations or analysis prior to acceptance, but the criticism can be solved by revising the discussion and the mathematical derivations and motivations. Most critically, a discussion on how to render the method "Bayesian" in a future study is needed, or to tone down the claim that the method would be Bayesian. The criterium used to stop the simulation must be stated (since Fig. S3 does not reveal any convergence). Apart from following the comments by Reviewer 3, please also revise Eq. 9., as the partial derivate of S with respect to S should be one. Maybe you need to use a different integration variable here?

> Appreciated; we believe we have addressed this by clarifying, revising the explanation of Eq. 9 to make it clearer that S is indeed the integration variable (and use parentheses to make the partial derivative usage clear, together with an extra step in what is now Eq. 10), we provide more context, and we have also modified the title to reflect that we do not have any axe to grind about whether the current formulation is strictly Bayesian or not. We fully agree on the *value* of pursuing an implementation where even the coupling strength is determined from data (ideally even in a spectrally dependent fashion), and we now cover this in the text as well as SI, but given Dr. Blau's limited time this is something we will need to find a new team member to focus on in future work.

Reviewer #1

The authors replied to almost all the questions and comments raised by three reviewers. We are satisfied about their replies to our previous comments. In particular, Figure 6, a new figure in the revised manuscript, seems to be meaningful to understand the behavior of relative entropy in the refinement. Before the publication of this manuscript, we suggest to correct the following points:
(1) add the label "C" in Figure 6. I can find only the labels "A" and "B" in Figure 6.
(2) Probably, the vertical axis "FSC_AVG@2.3A" seems to be wrong. 1/2.3 = 0.434. Figure 6b suggests that FSC value of this resolution (0.434) is almost zero. Please use the correct resolution in the vertical axis.

> Appreciated - done!

Reviewer #2

My concerns have been fully addressed in the revised version of the manuscript.

> Appreciated.

Reviewer #3 (our summary to avoid repeating equations from the PDF here)

Force constant k

As also commented on by the editor, this reviewer is still not convinced by Eqs. 1 - 4, whereby the force constant sneaks into framework.

> We agree that splitting the likelihood into force-constant and ”similarity” is a choice that is not formally grounded in a Bayesian framework, but rather a way of connecting existing practical approaches to our modeling approach, and on second thought the reviewer is probably right it's better to just keep things separate. We have updated Eq. 1 - 4 and surrounding text so that it is clearer how the force constant comes

into the framework, and no longer present it as necessary already in the first equation. We have also updated all derivations and make clear how free parameters in our modeling framework are related to force-constants in the established fitting frameworks.

Just as for any coupling or simulation, there is no unique way to determine when full convergence has been achieved, and in the current implementation it's even possible to keep running simulations until forces become so large that Gromacs stops the simulations, so that's actually what we did to show that there is no specific (cherry-picked) point after which the simulation result would deteriorate without the user knowing - although the simulation will of course no longer keep improving the RMSD to the (unknown) target towards the end. This too has been clarified in the text.

On the Dirichlet model

We have updated and corrected all derivations related to the Dirichlet model and removed our, admittedly clumsy, attempt to reason that keeping the free parameter is part of a Bayesian framework and streamlined the derivations instead, mentioning that indeed, a full Bayesian modeling of the unknown parameter via its conditional posterior would be a useful extension of this work. While we would have liked to pursue it already here, given that the first author now works in industry it has simply not been possible, so it is something we intend to pursue in the future with a new coworker.

Poisson model

In analogy to the Dirichlet model, we updated the derivation and removed the mention of the prior distribution for the model parameter r. We added the suggested conditional posterior distributions to the Supplementary Information, acknowledging reviewer three, for future work to continue from there.

On the topic of the magnitude of initial fitting forces, this will depend both on the fitting model used, the resolution of the map, and the initial molecular conformation. Cross-density will for instance have low forces far away from the target, or for low-resolution maps, but sometimes high forces close to high-resolution regions - and to first approximation the properties of cross-entropy are roughly the opposite. As for any simulation, they are simply sensitive when the initial problem is far from equilibrium, but for a true Bayesian model where we want to remove the parameter that could likely be handled with just limiting the initial strength.

Shortcomings of formal derivations

Many of the pointed out shortcomings (Eq. (7), equation on top of page 8) were now removed due to the fact that we do not attempt to integrate out the mixture parameters. We removed the not fully motivated attempt to integrate out the scaling parameter r by taking the expectation value of the potential, given p(r), rather than the correct way of integrating over the probabilities. We substantially toned down the claim that our method is a full Bayesian approach and replaced Bayes with maximum likelihood, and Bayes' approach with Bayes' theorem where appropriate. We still show how our method might be extended to fit a fully Bayesian framework in the Supplementary Information, thanks to the input from reviewer three.

Performance of the score

As described in the text the various methods have different properties, and we believe the claim that relative-entropy based score performs very well in the *low-noise scenario* holds (which we find interesting, since this appears to be the region many high-resolution new single particle structures are in). We have updated the abstract to explicitly mention the relative-entropy based potential, and to stress that all the potentials appear to benefit from the automated force scaling (and we believe users will find that valuable, even though it is not Bayesian).

Comparison with other methods

A comparison with other refinement methods using the same data is still missing.

Here we might disagree slightly about the best conceptual approach. We *strongly* prefer to perform a controlled comparison of different *equations/potentials* used to generate the force used in a refinement, and

Stockholm University

Stockholm May 25, 2023

how that influences the results. While it can be interesting from other perspectives to compare programs (e.g. how fast the refinement runs), such comparisons inevitably involve slight difference force fields (or implementations of the same force field), possibly different integrators, different ways to control statistical ensembles, preprocessing data, or possibly even bugs in one (or both) codes. All those things are interesting and important, but we do not believe it helps to involve them when trying to understand and compare properties of the refinement potentials *per se* - in our opinion it rather risks detracting from what we actually want to compare. However, at some point it is an excellent idea to perform a large-scale benchmark of practical refinement performance of several codes.

Minor points

In your simulations, which value do you use for s (scaling factor of the Dirichlet model)? Or is s identified with k and treated with force scaling?

Page 8: Striktly speaking, Eq. (5) is no equality, but only a proportionality.

The left parenthesis “(” in Eqs. (7) and (8) should be right of “log” (rather than left).

Thanks for spotting these; these equations have anyway been replaced, and we now explain on p.8 and p.10 that the force constant and scaling parameters end up being related as $k = k_B T s$, and we thus treat the through the force constant.

Sincerely,

/Erik Lindahl/
